# The effect of cyberbullying perpetration on depression in adolescents in western China: the mediating roles of self control and internet gaming addiction and the moderating role of meaning in life

**DOI:** 10.3389/fpsyg.2026.1749786

**Published:** 2026-03-11

**Authors:** Juan Jing, Jiaming Luo, Xin Hou, Yuxian Yan

**Affiliations:** 1 Psychological Health Center, Affiliated Hospital of North Sichuan Medical College, Nanchong, China; 2 Laboratory Psychology, Mental Health College, North Sichuan Medical College, Nanchong, China

**Keywords:** cyberbullying perpetration, depression, internet gaming addiction, meaning in life, self-control

## Abstract

**Background:**

This study amid to explore the relationship between cyberbullying perpetration and depression among adolescents in a western city of China. And verify the mediating role of self-control and Internet gaming addiction and the moderating role of meaning in life.

**Methods:**

A total of 8,209 adolescents were recruited through random sampling and cluster random sampling. Analyze the correlations among cyberbullying perpetration, self-control, Internet gaming addiction, meaning in life and depression. Mediation analyses were conducted to assess direct and indirect effects of cyberbullying perpetration on depression via self-control and Internet gaming addiction. Moderation analysis examined the direction of the moderating effect of meaning in life on the relationship between cyberbullying perpetration and self-control.

**Results:**

(1) There was a significant correlation between cyberbullying perpetration, self-control, Internet gaming addiction, meaning in life and depression. (2) Self-control and Internet gaming addiction played a significant partial mediating role in the relationship between cyberbullying perpetration and depression. The mediating effect consists of three indirect effects: Cyberbullying perpetration → self-control → depression (the mediating effect value is 0.159, *p* < 0.001), Cyberbullying perpetration → Internet gaming addiction → depression (the mediating effect value is 0.18, *p* < 0.001), and Cyberbullying perpetration → self-control → Internet gaming addiction → depression (the mediating effect value is 0.029, *p* < 0.001). (3) The interaction between cyberbullying perpetration and self-control was significant (*β* = −0.023, 95%CI −0.03 to −0.015, *p* < 0.01). Meaning in life plays a moderating role between cyberbullying perpetration and self-control. The stronger the meaning in life, the more obvious the negative effect of cyberbullying perpetration on self-control.

**Conclusion:**

(1) The research results indicate that cyberbullying perpetration is associated with higher symptoms of depression in adolescents. This association can occur directly or indirectly through reduced self-control and increased addiction to Internet gaming. (2) Adolescents with a stronger sense of meaning in life show a more pronounced decline in self-control after engaging in cyberbullying.

## 1 Introduction

With the development of social information and the convenience of Internet use, the use rate of digital products for young people has increased significantly. According to the 《2025 Measuring Digital Development: Facts and Figures》 report, global Internet penetration reached 74% in 2025, an increase of 3.3% relative to 2024. Domestic data released by People’s Daily indicates that China’s Internet penetration rate rose from 70.4 to 79.7% over the same period (People’s Daily, 2025). Moreover, the 6th National Survey on Internet Use among Minors reported that, as of 2023, 196 million Chinese minors were online, corresponding to an Internet penetration of 97.3% in this demographic. The age of children’s first contact with digital products and the use of the Internet also shows a trend of younger ages. Almost all adolescents aged 12–17 use the Internet (Kowalski et al., 2014). Internet use brings great convenience to life. However, early and unsupervised exposure to the Internet compromise adolescents’ psychological development. With the growing prevalence and importance of the Internet in adolescents’ daily lives, excessive Internet use among teenagers have become a significant public health concern (Marin et al., 2021). Existing research reports have shown that excessive use of the Internet leading to cyberbullying and Internet gaming addiction have become a public health issue that poses a threat to the physical and mental health of teenagers, and also increase the risk of depression among them (Chi et al., 2020; Rao et al., 2019; Ma and Sheng, 2023).

Cyberbullying perpetration is a new form of bullying that emerged with the rapid development of the Internet. It is defined as “…any behavior performed through electronic or digital media by individuals or groups that repeatedly communicates hostile or aggressive messages intended to inflict harm or discomfort on others. Cyberbullying is different from traditional bullying in its form and content (Tao et al., 2022). It is more difficult to control and predict, and often has a non-negligible impact on the mental health of adolescents. A comprehensive review of cyberbullying among adolescents and children shows that the prevalence rates of cyberbullying perpetration ranged from 6.0 to 46.3% (Zhu et al., 2021). At present, there are many studies on the mechanisms of depression caused by adolescent cyberbullying victims, but there are relatively few studies on the mechanisms of depression caused by cyberbullying perpetration among adolescents. We believe that exploring the mechanisms of cyberbullying perpetration is more meaningful than discussing the mechanisms of cyberbullying victims. Intervening in the cyberbullying perpetration of adolescents can not only fundamentally reduce the occurrence of cyberbullying victimization and alleviate its impact, but also improve the negative emotions of adolescents who engage in cyberbullying perpetration.

### 1.1 Cyberbullying perpetration and depression

Cyberbullying is an important predictor of emotional and behavioral problems in adolescents (Kim et al., 2018). Similar to victims of cyberbullying, perpetrators of cyberbullying may also face mental health issues (Bitar et al., 2023). A recent meta-analysis reported that the incidence of depression among those who engage in cyberbullying is 1.73 times higher than that of those who do not (Marciano et al., 2020). This association has a dual mechanism of action: on one hand, Ademiluyi et al. (2022) argue that the social exclusion and loss of peer support that often follow public identification as a bully erode protective social buffers, thereby precipitating depressive affect; on the other hand, Rinehart et al. (2020) also indicated that perpetrators who belittle victims undermine their own self-worth and self-esteem, thus aggravating psychological problems like anxiety and depression. More critically, sustained perpetration of cyberbullying may lead to self-harm and suicidal behaviors in adolescents (Hinduja and Patchin, 2019). Current research has shown a positive association between cyberbullying perpetration and depressive symptoms (Alrajeh et al., 2021; Wright and Wachs, 2023). Therefore, we propose Hypothesis 1: Cyberbullying perpetration can positively predict the occurrence of depression.

### 1.2 The mediating role of self-control

The sense of self-control refers to the perception of one’s own control (Rodin, 1989), the ability to change the development of events (Burger, 1986) and a psychological mechanism that enables individuals to face external pressure and resist distress (Tullett et al., 2015). Prior research has established the relationship between self-control and cyberbullying perpetration: low self-control is positively associated with aggressive and bullying behaviors (Li et al., 2023). Cyberbullying is characterized by multiple modalities, low implementation barriers, and limited accountability; these features intensify adolescents’ cyberbullying perpetration, and repeated cyberbullying perpetration progressively undermines their self-control capacity (Qing, 2024). Some bullies will make moral excuses to justify their behavior in order to reduce the guilt caused by bullying, which makes bullying more tolerable. This is equivalent to systematically destroying the self-control mechanism that conducts an internal moral review of behavior (Runions and Bak, 2015; Walrave and Heirman, 2011). As self-control decreases, individuals find it difficult to regulate their emotions when facing stressful events, which can easily lead to negative psychology and an increased risk of depression (Zeidi et al., 2020). The following tentative hypothesis 2 was developed. H2: Cyberbullying perpetration can predict the occurrence of depressive mood through the mediating role of self-control.

### 1.3 The mediating role of internet gaming addiction

Internet gaming addiction refers to the individual’s uncontrolled, dependent, compulsive use of Internet gaming. With the widespread adoption of smartphones and the increasing age of adolescents, they spend more time online. A large number of studies have shown that Internet gaming addiction has negative impacts on the physical health, mental health, interpersonal relationships, and academic performance of adolescents (De Pasquale et al., 2020; Ye et al., 2025; King et al., 2020). Multiple studies have reported a positive correlation between cyberbullying and Internet gaming addiction. Participating in cyberbullying increases the probability of developing Internet gaming addiction (Ozyürek et al., 2023; Xiang et al., 2022). Firstly, the disinhibitory effect of the internet—due to its anonymity and the absence of traditional social constraints, individuals are more likely to engage in disinhibited behaviors online, which can lead to addiction to online games and create conditions for cyberbullying (Yang et al., 2025). Secondly, meta-analysis also pointed out that bullying and prolonged Internet use are risk factors for Internet gaming addiction among children and teenagers (Gao et al., 2022). Higher frequency of cyberbullying perpetration heightens vulnerability to Internet gaming addiction, which is consistent with the GAM theory. The GAM theory model indicates that repeated exposure to or contact with violence will enhance an individual’s aggression, making them more addicted to online violent gaming (Anderson and Carnagey, 2004). Cyberbullying is an immoral behavior. In order to avoid the emergence of self-blame and self-punishment, cyberbullies will also use the game to paralyze themselves or release emotions in the game. However, long-term addiction to Internet gaming will affect individuals’ real-life interpersonal relationships, academic performance, etc., and may lead to psychological problems such as depression. Therefore, we believe that bullying behavior increases the risk of adolescents becoming addicted to online games, which in turn may indicate the onset of depressive symptoms. With the above analysis, hypothesis 3 was formulated. H3: cyberbullying perpetration can positively predict a depressive mood through the mediating effect of Internet gaming addiction.

### 1.4 The chain mediating effect of self-control and online gaming addiction

Adolescence is a critical period for psychosocial development, and cyberbullying exerts pronounced negative effects on youths’ mental health. Previous studies reported that moral disengagement is significantly associated with cyberbullying (Kowalski et al., 2014). By activating the moral disengagement mechanism, individuals can temporarily suspend their moral standards, allowing harmful acts to be committed without remorse or negative self-sanction, while maintaining a positive view of themselves, thereby reducing the sense of guilt for committing bullying behavior (Bussey et al., 2024). Meanwhile, the “sense of achievement” derived from bullying will activate the brain reward mechanism, leading to a decline in an individual’s self-control. When the individual’s self-control weakens, their self-discipline also diminishes. The individual is more likely to become engrossed in the pleasure derived from online gaming, leading to an extended period of online use. However, prolonged internet addiction can impair an individual’s real-life interpersonal relationships and academic performance, ultimately increasing study pressure, deteriorating interpersonal communication, and elevating the risk of depression (Ye et al., 2023; Erdem and Sezer Efe, 2022). As a result, hypothesis 4 was assumed. H4: Self-control and Internet gaming addiction play a chain mediating role between cyberbullying perpetration and depression.

### 1.5 Moderating role of the sense of meaning in life

Steger et al. (2015) defined the “meaning in life” as an individual’s realization of life’s value and meaning of life, along with the clarity of life goals and aspirations. Previous studies indicate that meaning in life is positively correlated with self-control and negatively correlated with depression (Liu et al., 2022; Glaw et al., 2016), It has a positive predictive effect on individual well-being and a positive protective effect on mental health, and can buffer the impact of negative events (Van Tongeren and Green, 2010). MacKenzie and Baumeister (2014) posits that the meaning of life serves multiple functions, including helping regulate negative emotions, coordinate individual behavior, and enhance self-control. Consequently, we hypothesize that the sense of meaning in life may regulate the relationship between cyberbullying perpetration and self-control (H5).

**Figure 1:**
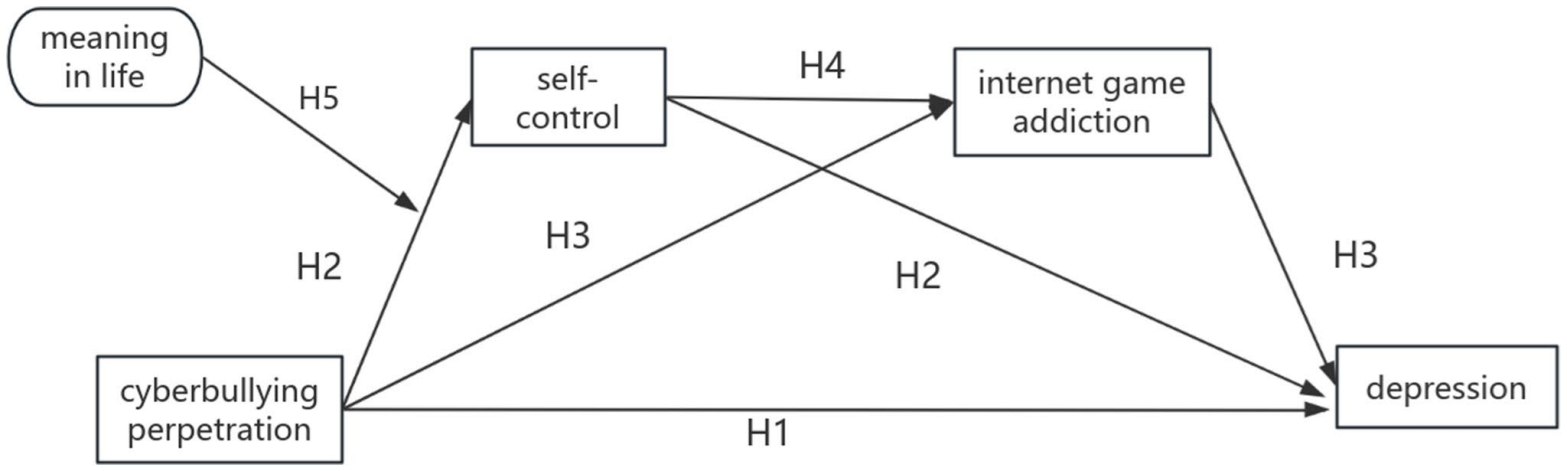
The proposed model about cyberbullying perpetration among adolescents in this study. Hypothesis. H1: Cyberbullying perpetration can positively predict the occurrence of depression. H2: Self-control mediated the relationship between cyberbullying perpetration and depression. H3: Internet gaming addiction mediated the relationship between cyberbullying perpetration and depression. H4: Self-control and Internet gaming addiction mediated the relationship between cyberbullying perpetration and depression. H5: Meaning in life moderated the effect of cyberbullying perpetration and self-control.

In summary, this study sought to construct a moderated chain mediation model and proposed the following hypotheses (see Figure 1).

## 2 Methods

### 2.1 Study design, participants, and setting

This study employed a cross-sectional survey design. First, randomly selected two county-level administrative districts from the six districts governed by Nanchong City in Sichuan Province. Next, within the selected districts, conduct a lottery for all primary and secondary schools and randomly select 30% of these schools. All students in grades 4–12 from the selected schools will be included in the research sample. Inclusion criteria: (1) aged 10–19 years old; (2) no clinical diagnosis of intellectual disability; (3) having the ability to independently complete the questionnaire. Exclusion criteria: (1) students or guardians who refuse to participate; (2) a missing rate of key questionnaire variables exceeding 15%. All participants and guardians signed informed consent and were included in the study. The research emphasizes the rights of voluntary participation.

The participants were promised to keep the data confidential and emphasized that the receipt data would only be used for scientific research purposes. The survey period was from April to May 2025, and the data was collected through the Wenjuanxing platform.^1^ The questionnaire distribution was completed by research assistants who had received unified training and collaborated with the head teacher. The research assistants are composed of psychiatrists from the Affiliated Hospital of North Sichuan Medical College and postgraduate and undergraduate students from the School of Mental Health of North Sichuan Medical College. Before participating in the research, each research assistant needs to receive training in professional knowledge and questionnaire content and pass relevant tests. Standardized instructions were provided to explain the filling norms to ensure independent answer. The average completion time of the questionnaire was within 30 min. To ensure data reliability, only responses completed within 200–2,400 s are considered valid. Responses that are too fast or too slow are considered invalid. The questionnaire design requires that all questions be answered before submission. The study protocol was approved by the Ethics Committee of the North Sichuan Medical College (Project Number: NSMC [2024]014).

The RAOSOFT online calculator was used to calculate the sufficient statistical power of the sample size. On the basis of 71,772 college students^2^^,^^3^ a 3% margin of error, a 95% confidence level, and a 50% response distribution, the sample size should not be less than 1,052. During the study period, a total of 10,046 questionnaires were ultimately collected. After deleting the invalid ones, 8,209 valid questionnaires were obtained. The average age of the students was 13.62 years (SD = 2.32), and 52.2% were male.

### 2.2 Measures

#### 2.2.1 Demographics

A self-designed questionnaire was used to collect the demographic information of the participants, including age, gender, ethnicity, grade, dwelling environment, experience of being left behind.

#### 2.2.2 Cyberbullying perpetration and victimization

Cyberbullying (perpetration and victimization) was measured using the brief adaptation of the Electronic Bullying Questionnaire (EBQ) (Moore et al., 2012). The EBQ includes both the cyberbullying perpetration subscale (4 items, e.g., “Have you ever made fun of others on the Internet, or teased them in a hurtful way?”) and cyberbullying victimization subscale (4 items, e.g., “Has anyone ever made fun of you or teased you in a hurtful way on the Internet?”). Participants responded on a five-point Likert scale ranging from one (it has not happened in the past couple of months) to five (several times a week). These two subscales were scored separately. Were calculated, with higher mean scores representing higher frequencies of cyberbullying perpetration and cyberbullying victimization. The EBQ has shown adequate psychometric properties in Chinese adolescents, The Cronbach’s alphas were 0.80 for bullying subscale and 0.78 for the victimization subscale (Tian et al., 2018). This study only analyzes the dimension of cyberbullying perpetration and the Cronbach’s *α* coefficient was 0.86.

#### 2.2.3 Brief self-control scale (BSCS)

This study used the seven-item new brief self-control scale (BSCS) as a measurement of self-control ability (Morean et al., 2014). This scale encompasses two dimensions: Self-Discipline (3 items, e.g., “I am good at resisting temptation”) and Impulse Control (4 items, e.g., “I do certain things that are bad for me if they are fun”). It utilizes a Likert five-point rating scale, ranging from 1 (completely inconsistent) to 5 (completely consistent). Among the items, items 2, 4, 6, and 7 are reverse-scored. We calculated the average score of the seven items: the higher the score, the higher the level of self-control. The Chinese version has shown adequate psychometric properties in Chinese adolescents. The Cronbach’s *α* coefficient of the total scale was 0.83, while the *α* coefficients for the two dimensions of self-discipline and impulse control were 0.85 and 0.86, respectively (Luo et al., 2021). In this study, the Cronbach’s *α* coefficient for the Chinese version of the BSCS was 0.7.

#### 2.2.4 Ten-item internet gaming disorder test (IGDT-10)

This scale was compiled by Király et al. (2019) and revised by Yang Haibo. The scale has a total of 10 items (e.g., “when you were not playing, how often have you fantasized about gaming, thought of previous gaming sessions, and/or anticipated the next gaming?”). Participants were asked to rate items on a three-point Likert scale labeled 0 (never), 1 (sometimes), and 2 (often). In order to match the dichotomous structure of IGD in DSM-5, ‘never ‘and ‘sometimes ‘can be re-encoded as 0. Given that questions 9 and 10 are related to the same criterion, they were combined in the scoring, that is, answering “often” on both Item 9 or 10 (or both items) scores only 1 point. The composite score of IGDT-10 ranged from 0 to 9, with higher scores indicating more IGD symptom. Cronbach’s alpha of the scale was 0.68, while Guttman’s Lambda-2 value was 0.69. The Chinese version of IGDT-10 showed adequate psychometric properties in adolescents. The Cronbach’s *α* coefficient was 0.85 (Chiu et al., 2018). In this study, the Cronbach’s *α* coefficient for the Chinese version of IGDT-10 was 0.9.

#### 2.2.5 Meaning in life questionnaire (MLQ)

This scale was compiled by Steger et al. (2006) and revised by Wang Xin qiang. The scale consists of 10 items, including two dimensions of Search for meaning (MLQ-S) (5 items, e.g., “I am seeking a goal or mission in my life”) and Presence of meaning (MLQ-P) (5 items, e.g., “My life has no definite purpose.”). Participants were asked to rate items on a seven-point Likert scale, ranging from 1 (completely inconsistent) to 7 (completely consistent). Among the items, items 2 is reverse-scored. Where higher scores indicate higher levels of meaning in life. The Chinese version has good reliability and validity among middle school students. The Cronbach’s *α* coefficient of the total scale was 0.83, while the *α* coefficients for the two dimensions of seeking meaning and being meaning were 0.842 and 0.828, respectively (Wang, 2013). In this study, the Cronbach’s *α* coefficient for the Chinese version of MLQ was 0.83.

#### 2.2.6 Patients ‘health questionnaire depression scale-9 item (PHQ-9)

This scale was compiled by Kroenke et al. (2001) and it was used quantify the severity of depression within the past 2 weeks. This questionnaire has higher reliability (Cronbach’s *α* = 0.86–0.89) in various clinical settings. The scale consists of 9 items (e.g., “Little interest or pleasure in doing things.”). It utilizes a Likert four-point rating scale, ranging from 0 (not at all) to 3 (nearly every day). A PHQ-9 total score greater than 5 indicates the presence of depression. This questionnaire has demonstrated good reliability and validity in Chinese population (Yin et al., 2022). In this study, the Cronbach’s *α* coefficient of the scale was 0.91.

### 2.3 Statistical analyses

Since the data was obtained based on questionnaire survey. To detect and control potential common method biases, we included reverse items in the “Meaning in Life Questionnaire” and the “Brief Self-Control Scale” during the design of the questionnaire. This study used IBM® SPSS® Statistics 26.0 with a plug-in unit Process V4.0 to manage and analyze the data. Before analysis, if the data for a key variable is missing by more than 15%, it should be deleted. The specific steps are as follows: (1) Harman single factor test was used to evaluate the common method bias; (2) Calculate the mean, standard deviation and Pearson correlation coefficient matrix for each variable; (3) Mediation and Moderation Analysis with PROCESS (Hayes, 2013): Models 6 and 83 in the PROCESS v4.0 plug were used to test the mediating and moderating effects; The primary tests include: the direct effect of cyberbullying perpetration on depression; the mediating role of self-control and Internet gaming addiction in this relationship; the moderating effect of meaning in life on the relationship between cyberbullying perpetration and self-control. (4) The Bootstrap method was used to repeatedly sample 5,000 times, and the 95% confidence interval (95% CI) did not contain zero as the criterion for significant effects.

### 2.4 Common method bias test

We used Harman’s single-factor test to test the common method bias. The results showed that there were seven factors with eigenvalues greater than 1, and the contribution rate of the first factor was 22.8%, far lower than the critical value of 40% (Tang and Wen, 2020). These results indicated that there was no common method deviation in this study.

## 3 Results

### 3.1 Descriptive statistics and correlation analysis of each variable

The descriptive statistics and correlation analysis results of each variable are shown in Table 1. The results showed that cyberbullying perpetration was significantly negatively correlated with self-control and meaning in life, and significantly positively correlated with Internet gaming addiction and depression. Self-control was significantly positively correlated with meaning in life, and significantly negatively correlated with Internet gaming addiction and depression. The sense of meaning in life was negatively correlated with Internet gaming addiction and depression. Internet gaming addiction was significantly positively correlated with depression.

**Table 1:** Descriptive statistics and correlation analysis of each variable (*n* = 8,209).

Variable	M ± SD	1	2	3	4	5
1. Cyberbullying perpetration	4.2 ± 1.012	1				
2. Self-control	23.66 ± 5.246	−0.121^**^	1			
3. Meaning in life	47.98 ± 13.03	−0.065^**^	0.282^**^	1		
4. Internet gaming addiction	0.33 ± 13.081	0.226^**^	−0.277^**^	−0.052^**^	1	
5. Depression	12.74 ± 4.711	0.189^**^	−0.402^**^	−0.146^**^	0.307^**^	1

*p < 0.05, **p < 0.01, ***p < 0.001.

Regarding demographic variables, the following continuous variables are significantly correlated with the main research variables to a certain extent: age, gender, grade, place of residence, experience of being left behind. These factors will be incorporated as control variables, with categorical variables undergoing dummy variable coding.

### 3.2 Chain mediating effect test

After controlling for the effects of control variables. A mediation effect test was conducted, using cyberbullying perpetration as the independent variable, depression as the dependent variable, and self-control and Internet gaming addiction as the mediating variables.

**Figure 2:**
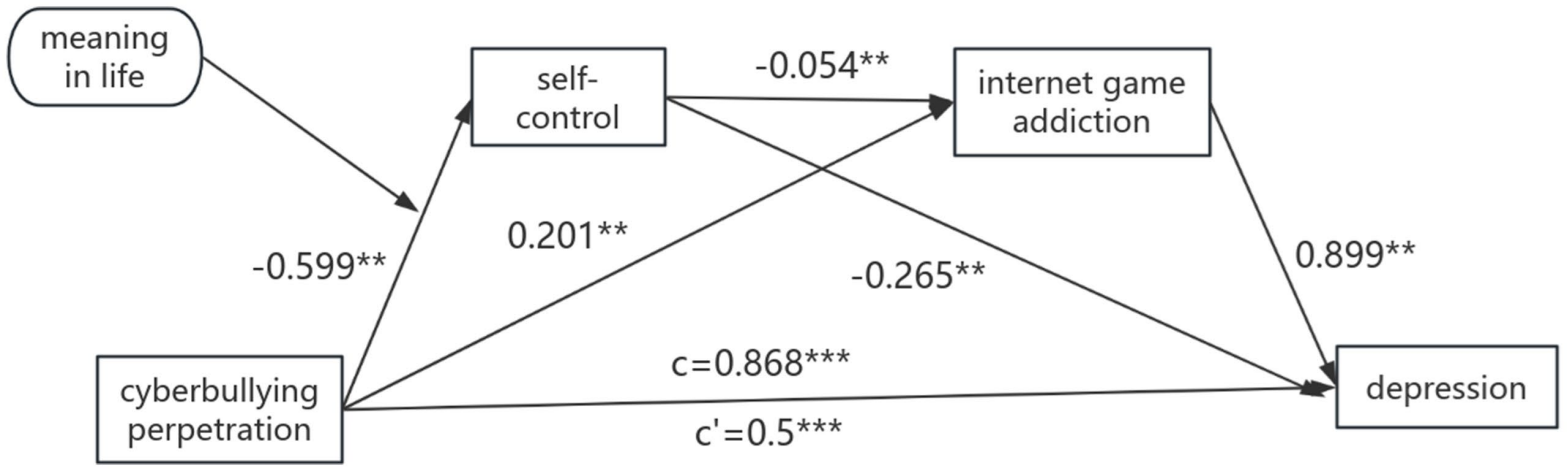
Mediation models of cyberbullying perpetration and depression through self-control and Internet gaming addiction. Cyberbullying perpetration is the independent variable, depression is the dependent variable, Internet gaming addiction and self-control are the mediating variable. ***p < 0.001;**p < 0.01; c = total effect; c′ = direct effect. All variables were standardized.

The results of bias-corrected percentile bootstrap analysis revealed significant indirect effects of self-control and Internet gaming addiction on the relationship between cyberbullying perpetration and depression (Figure 2 and Table 2). The total effect of cyberbullying perpetration on depression was 0.868 (SE = 0.049, *p* < 0.001, boot 95% BC CI [0.772, 0.964]). It shows that cyberbullying perpetration significantly positively predicts depression, which provides support for Hypothesis 1. The total indirect effect of cyberbullying perpetration on depression was 0.368 (SE = 0.035, *p* < 0.001, boot 95% BC CI [0.303, 0.44]). The ration of indirect effect of cyberbullying perpetration on depression to the total effect was 42.4%, indicating that self-control and Internet gaming addiction played partial mediating effects, which provides support for Hypothesis 4.

**Table 2:** Testing the mediating effect of cyberbullying perpetration on depression.

Effect	Path relationship	Effect	Boot SE	Boot LLCI	Boot ULCI	Effect proportion (%)
Direct effect	Cyberbullying perpetration → depression	0.5	0.046	0.41	0.59	57.6
Indirect effect	Cyberbullying perpetration → self-control → depression	0.159	0.017	0.129	0.194	18.32%
	Cyberbullying perpetration → Internet gaming addiction → depression	0.18	0.027	0.13	0.237	20.74%
	Cyberbullying perpetration → self-control → Internet gaming addiction → depression	0.029	0.004	0.022	0.038	3.34%
Total indirect effect		0.368	0.035	0.303	0.44	42.40%
Total effect		0.868	0.049	0.772	0.964	

The path 1: The indirect effect of cyberbullying perpetration → self-control → depression path was significant, and the effect value was 0.159 (boot 95% BC CI [0.129, 0.194]), Path 2: The indirect effect of cyberbullying perpetration → Internet gaming addiction → depression path was significant, and the effect value was 0.18 (boot 95% BC CI [0.13, 0.237]). Path 3: The indirect effect of cyberbullying perpetration → self-control → Internet gaming addiction → depression path was significant, and the effect value was 0.029 (boot 95% BC CI [0.022, 0.038]). All the three paths were significant because their 95% CI did not include zero. The mediating effects of the three paths accounted for 18.32, 20.74, and 3.34% of the total indirect effects, respectively. The results verified the hypotheses regarding the direct and indirect effects of cyberbullying perpetration on depression, and the partial mediating effects of self-control and Internet gaming addiction, which provides support for Hypothesis 2 and 3.

### 3.3 Testing for the moderated mediating effect of meaning in life

**Figure 3:**
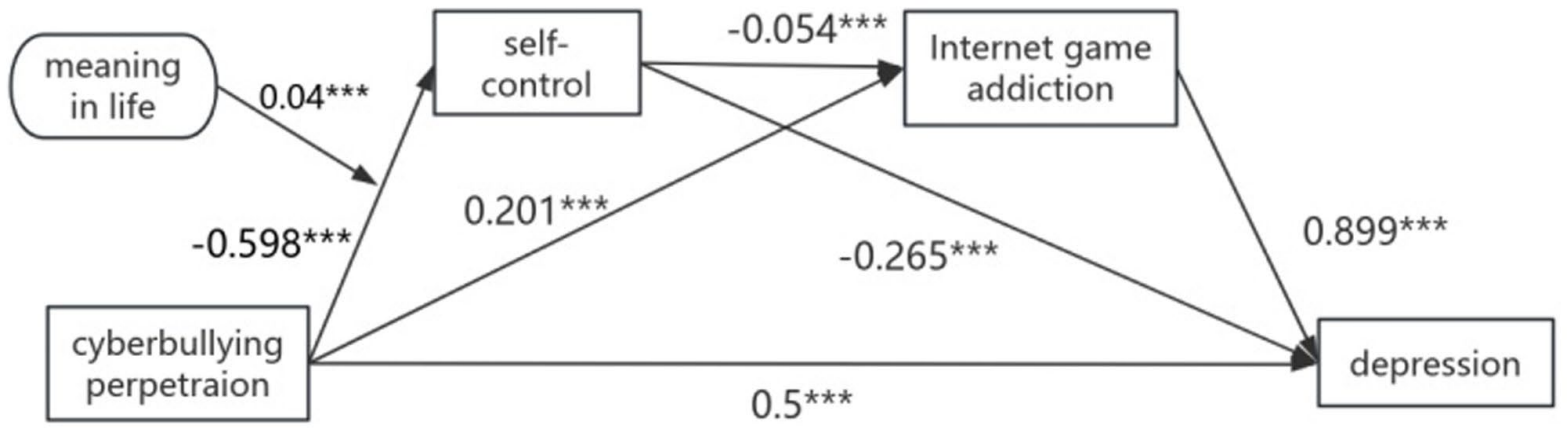
The moderated mediation model.

We used Model 83 in the PROCESS macro to examine the moderating effect of meaning in life on between cyberbullying perpetration and self-control. To prevent multicollinearity, we standardized all predictive variables. Figure 3 shows that the interaction between cyberbullying perpetration and meaning in life has a significant effect on self-control (*β* = −0.025, SE = 0.004, *p* < 0.01). Hypothesis 5 was supported.

**Figure 4:**
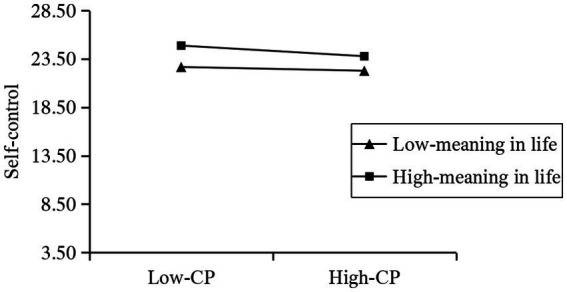
Meaning in life moderates the relationship between Cyberbullying perpetration and Self-control. CP, Cyberbullying perpetration.

In order to explore how the sense of meaning in life affects the relationship between the perpetration of cyberbullying and self-control, this study conducted a simple slope test (see Figure 4 and Table 3). Simple slope tests revealed that the association between cyberbullying perpetration and self-control was stronger for adolescents with high meaning in life (βsimple = −0.924, SE = 0.086, 95% CIs = [−1.092, −0.757]) than those with low meaning in life (βsimple = −0.272, SE = 0.067, 95% CIs = [−0.402,-0.141]).

**Table 3:** Comparison of inter-group differences in the moderating effects of different levels of sense of meaning in life.

Moderator variable	Effect	Boot SE	Boot LLCI	Boot ULCI
Low-scoring group (M − 1SD)	−0.272	0.067	−0.402	−0.141
Middle group (M)	−0.598	0.056	−0.707	−0.489
High-score group (M + 1SD)	−0.924	0.086	−1.092	−0.742

## 4 Discussion

Unlike most prior studies, which mainly focused on adolescent victims of cyberbullying, this study selects primary and secondary school students from multiple schools in a western Chinese city as its research subjects. From the perspective of adolescents who engage in cyberbullying, with self-control and Internet gaming addiction as mediators and the sense of meaning in life as a moderator, a moderated chain mediation model was developed. This model explores the impact and mechanisms of cyberbullying perpetration on adolescent depression. The results of this study provide empirical support for all the proposed hypotheses. The results show that cyberbullying behavior directly and indirectly affects the depressive mood of adolescents through self-control and Internet gaming addiction, and the sense of meaning in life plays a moderating role in it. These findings highlight the crucial role of cyberbullying behavior, self-control, online gaming addiction and a sense of meaning in life in the development of depression emotion among adolescents.

Research results indicate cyberbullying perpetration would positively predict the occurrence of depression. Increased frequency of engagement in cyberbullying correlates with a higher risk of developing depression. This is consistent with previous studies (Hinduja and Patchin, 2010; Calpbinici and Tas Arslan, 2019). This discovery can be explained from the following aspects: First, role inversion indicates that which those involved in one role are more likely to become involved in the other role. For example, victimization may subsequently become perpetrators. Individuals often experience depressive moods following bullying. However, when they transform into perpetrators, their emotions cannot be truly relieved. On the contrary, adolescents may experience feelings of depression due to isolation or rejection by peers, as a negative consequence of bullying behavior. Secondly, although research reports indicate that adolescents who engage in cyberbullying tend to exhibit higher levels of violence (Sari and Camadan, 2016), aggression (Ybarra and Mitchell, 2004), lack of empathy (Steffgen et al., 2011), are prone to anti-social behavior (Sticca et al., 2013) and an increased tendency for self-harm (Hinduja and Patchin, 2010); However, not all bullies fit this description. Some bullies may feel regret for their involvement in bullying due to moral condemnation, which can exacerbate feelings of depression. Thus, this suggests that while we pay attention to the phenomenon of cyberbullying perpetration among adolescents, we must also recognize the emotional and behavioral issues underlying such bullying behaviors.

This study shows that there is a negative correlation between engaging in cyberbullying and self-control, as well as a negative correlation between self-control and depression. Specifically, adolescents who frequently engage in cyberbullying often exhibit lower self-control, and decreased self-control further increases the risk of depression. This finding is consistent with previous research (Mason, 2008)^.^ This study also found that self-control plays a mediating role between cyberbullying perpetration and depression. This finding enriches the mechanism of the impact of cyberbullying perpetration on teenagers’ depression. The reasons are as follows: On one hand, the anonymity of the internet reduces the cost of bullying behavior, making such actions easier to occur. Additionally, the lag in online regulation separates the act of cyberbullying from its consequences, which can weaken self-restraint and increase impulsivity. On the other hand, some bullies experience moral disengagement, minimizing the harm caused by their bullying actions and maximizing the reduction of their own responsibility for the consequences, thereby rationalizing their behavior and leading to a decrease in self-control. Self-control is the individual’s ability to manage emotions, behaviors, and impulses; it is a limited resource (Baumeister et al., 1998). As it continues to be consumed, individuals may easily experience emotional dysregulation and a sense of loss of control, which can trigger depression. Self-control is an important factor in predicting the likelihood of depression among adolescents and should be emphasized. Future research could focus on reducing depression levels in adolescents by enhancing self-control, which is expected to become a new intervention direction.

The results of this study show that cyberbullying perpetration is positively correlated with Internet gaming addiction, Internet gaming addiction is positively correlated with depression, and Internet gaming addiction mediates the influence of cyberbullying perpetration on depression. This suggests that cyberbullying perpetration can positively predict depression through Internet gaming addiction. Previous studies have shown that individuals who engage in cyberbullying frequently spend substantial time online and are readily drawn to violent or stimulant-based gaming (Brand et al., 2019). The enjoyment derived from gaming activates the dopamine reward pathway, encouraging individuals to perpetually seek stimulation. Particularly, adolescents have underdeveloped prefrontal cortex, making them more susceptible to immediate gratification from gaming and thus more prone to excessive gaming behavior. Moreover, the sense of power and victory in games is similar to the sense of domination in cyberbullying perpetration, which makes teenagers prone to addiction to the virtual world of games. When adolescents become addicted to online games, they experience reduced social support, increased loneliness, diminished self-worth, and decreased sleep quality, making them more susceptible to negative emotions and events, thereby increasing the risk of depression (Li et al., 2021; Xie and Tang, 2024). Additionally, after discontinuing gaming, dopamine levels exhibit a “relative trough,” and the contrast effect of emotions makes real-life activities appear more tedious, potentially inducing anhedonia. This suggests that adolescents who exhibiting cyberbullying behaviors can be screened for comorbid Internet gaming addiction. Perhaps the window period when “bullying occurring but internet gaming addiction not yet severe” could serve as a breakthrough to reduce the risk of depression.

The study also found that self-control and Internet gaming addiction play a partially chain-mediated role between cyberbullying perpetration and depression. Cyberbullying perpetration not only directly leads to depression, but more importantly, it reduces individuals’ self-control by depleting their self-regulatory resources, which prompts them to rely on Internet gamings as a coping mechanism, ultimately leading to addiction. This phenomenon can be attributed to two main reasons. On one hand, cyberbullies tend to exhibit higher impulsivity and poorer emotional regulation abilities (Ybarra and Mitchell, 2007; Donahue et al., 2014). First, cyberbullying perpetrators exhibit heightened impulsivity and impaired emotional regulation (Steger et al., 2006; Wang, 2013), which makes them more likely to turn to the online world for comfort when faced with frustration. The immediate rewards and information overload provided by Internet environments weaken their self-control, leading to prolonged Internet use and creating conditions for Internet gaming addiction. Second, cyberbullying perpetration consumes adolescents’ self-regulatory resources, making them more inclined toward low-difficulty, easily controllable online activities. The instant feedback, variable ratio reinforcement, and anonymous settings of online games precisely meet the demands for “low investment - high return.” Additionally, individuals with diminished self-control are more susceptible to indulgence in instant gratification (such as binge eating, internet use, or substance abuse). While these short-term escapisms provide momentary relief, they consume time and energy needed for pursuing long-term goals, gradually eroding self-efficacy. When real-life achievements become difficult to attain, feelings of helplessness accumulate, ultimately resulting in depressive emotions.

The study also found that the sense of meaning in life has a negatively moderating effect between cyberbullying perpetration and self-control. Specifically, individuals with a stronger sense of meaning in life exhibit a more pronounced decline in self-control after engaging in cyberbullying perpetration, which is inconsistent with previous research findings (Li et al., 2023). This can be interpreted as individuals with a high sense of meaning typically possess clearer values, empathy, and a strong moral sense, leading them to pursue a purposeful and meaningful life. When individuals engage in cyberbullying, such actions conflict sharply with their intrinsic values (such as kindness, respect, and compassion). This internal conflict depletes psychological resources, causing significant “ego depletion” and reducing impulse control, which can lead to negative emotional states. Conversely, individuals with unclear moral standards or a lack of meaning in life are less prone to experience similar psychological burdens, reducing their likelihood of depression. Moreover, the present study revealed that both individuals with strong and weak senses of meaning in life reported diminished self-control as cyberbullying perpetration increased. This demonstrates that cyberbullying perpetration, as an aggressive behavior, can profoundly impact anyone’s psychological and behavioral well-being. The strength of one’s sense of meaning in life determines the rate of psychological resource depletion, rather than its endpoint.

## 5 Conclusion

(1) The findings demonstrate that cyberbullying perpetration can directly promote depression. Self-control and Internet gaming addiction play a partial mediating role between cyberbullying perpetration and depression, and meaning in life moderates the association between cyberbullying perpetration and self-control. (2) Regardless of whether the sense of meaning in life is strong or weak, adolescents who engage in cyberbullying perpetration will exhibit a decline in self-control, especially for those with a strong sense of meaning in life, where the decline in self-control is more pronounced.

## 6 Limitations and future directions

This study has several strengths, including a large, school-based sample, the use of validated instruments, and the examination of a moderated chain mediation model that integrates behavioral and psychological mechanisms. This study focuses on cyberbullying perpetration rather than the victims, providing a new perspective for explaining the underlying mechanism of depression induced by cyberbullying perpetration. By taking the sense of meaning in life as a moderating variable, this study clarifies how the implementation of cyberbullying perpetration affects the decline of self-control among adolescents. These findings provide directions for thinking about the prevention and intervention of depression among teenagers with cyberbullying perpetration.

Despite several strengths, several limitations of this study should be recognized. (1) This study focuses on the cyberbullying perpetration among adolescents. However, cyberbullying perpetration is affected by various factors, such as social and environmental background, demographic situations, etc. Future research can explore the underlying mechanisms of cyberbullying perpetration from more perspectives, providing intervention directions to reduce cyberbullying perpetration. (2) Although this study is based on a certain theoretical basis, it employs a cross-sectional design, which can only confirm correlations rather than establish the temporal sequencing of cyberbullying behaviors, mediating variables, and depression. Future research could conduct longitudinal studies to investigate the relationship between cyberbullying perpetration at different points in time and depression. (3) The data in this study are based on self-report form of Internet platform. Reliance on self-reported data may potential introduce a systematic bias, and adolescents might deliberately conceal or misremember their cyberbullying experiences when questioned about this sensitive topic. Future studies could further incorporate psychological imaging indicators, combined with brain functional imaging changes, to reveal deeper impacts of cyberbullying perpetration. (4) Recruiting participants from a single city in western China may introduce region-specific cultural or contextual influences, thereby limiting the external validity of the findings for other geographic or cultural settings. Future research should enroll participants from different regions and cultures to enhance generalizability.

## Data Availability

The raw data supporting the conclusions of this article will be made available by the authors without undue reservation.

## Footnotes

1
https://ww.wjx.cn/

2 Available at: https://www.xichong.gov.cn/zwgk/fdzdgknr/tjsj/202407/t20240703_1990654.html.

3 Available at: https://www.langzhong.gov.cn/zwgk/zdlygk/ywjy/202401/t20240111_1926815.html.
